# Larissa Heart Failure Risk Score and Mode of Death in Acute Heart Failure: Insights from REALITY-AHF

**DOI:** 10.3390/jcm12113722

**Published:** 2023-05-28

**Authors:** Andrew Xanthopoulos, Angeliki Bourazana, Yuya Matsue, Yudai Fujimoto, Shogo Oishi, Eiichi Akiyama, Satoshi Suzuki, Masayoshi Yamamoto, Keisuke Kida, Takahiro Okumura, Grigorios Giamouzis, John Skoularigis, Filippos Triposkiadis, Takeshi Kitai

**Affiliations:** 1 Department of Cardiology, University Hospital of Larissa, 41110 Larissa, Greece; 2 Department of Cardiovascular Medicine, Juntendo University Graduate School of Medicine, Tokyo 113-0033, Japan; 3 Department of Cardiology, Himeji Cardiovascular Center, Himeji 670-8560, Japan; 4 Division of Cardiology, Yokohama City University Medical Center, Yokohama 232-0024, Japan; 5 Department of Cardiovascular Medicine, Fukushima Medical University, Fukushima 960-1295, Japan; 6 Cardiovascular Division, Faculty of Medicine, University of Tsukuba, Tsukuba 305-8577, Japan; 7 Department of Pharmacology, St. Marianna University School of Medicine, Kawasaki 216-8511, Japan; 8 Department of Cardiology, Nagoya University Graduate School of Medicine, Nagoya 466-8550, Japan; 9 Department of Cardiovascular Medicine, Kobe City Medical Center General Hospital, Kobe 650-0047, Japan; 10 Department of Cardiovascular Medicine, National Cerebral and Cardiovascular Center, Osaka 564-8565, Japan

**Keywords:** Larissa heart failure risk score, mode of death, sudden cardiac death, mortality

## Abstract

Patients with heart failure (HF) patients may die either suddenly (sudden cardiac death/SCD) or progressively from pump failure. The heightened risk of SCD in patients with HF may expedite important decisions about medications or devices. We used the Larissa Heart Failure Risk Score (LHFRS), a validated risk model for all-cause mortality and HF rehospitalization, to investigate the mode of death in 1363 patients enrolled in the Registry Focused on Very Early Presentation and Treatment in Emergency Department of Acute Heart Failure (REALITY-AHF). Cumulative incidence curves were generated using a Fine–Gray competing risk regression, with deaths that were not due to the cause of death of interest as a competing risk. Likewise, the Fine–Gray competing risk regression analysis was used to evaluate the association between each variable and the incidence of each cause of death. The AHEAD score, a well-validated HF risk score ranging from 0 to 5 (atrial fibrillation, anemia, age, renal dysfunction, and diabetes mellitus), was used for the risk adjustment. Patients with LHFRS 2–4 exhibited a significantly higher risk of SCD (HR hazard ratio adjusted for AHEAD score 3.15, 95% confidence interval (CI) (1.30–7.65), *p* = 0.011) and HF death (adjusted HR for AHEAD score 1.48, 95% CI (1.04–2.09), *p* = 0.03), compared to those with LHFRS 0,1. Regarding cardiovascular death, patients with higher LHFRS had significantly increased risk compared to those with lower LHFRS (HR 1.44 adjusted for AHEAD score, 95% CI (1.09–1.91), *p* = 0.01). Lastly, patients with higher LHFRS exhibited a similar risk of non-cardiovascular death compared to those with lower LHFRS (HR 1.44 adjusted for AHEAD score, 95% CI (0.95–2.19), *p* = 0.087). In conclusion, LHFRS was associated independently with the mode of death in a prospective cohort of hospitalized HF patients.

## 1. Introduction

Heart failure (HF) is a lethal syndrome affecting 38 million adults globally [1]. Due to the senescence and expansion of the global population, its prevalence continues to rise [2]. Patients with HF suffer a progressive decline in their functional and intellectual capacity, while the risk of sudden cardiac death (SCD) is low. Since its designation as an emerging pandemic in 1997, HF has attracted a host of studies with the purpose of corroborating our mechanistic understanding of the syndrome. Nevertheless, the burden of mortality and hospitalizations varies significantly among the different HF groups [3]. Disparate is also a mode of death where some patients die suddenly while others die from disease progression, such as pump failure or non-cardiovascular death [4]. The evident heterogeneity in the clinical profiles of HF necessitates a profound understanding of the factors associated with the mode of death in HF.

The Larissa Heart Failure Risk Score (LHFRS) is a practical risk stratification model derived from three factors (history of hypertension (yes = 0, no = 2); history of coronary artery disease/myocardial infarction (yes = 1, no = 0); and red blood cell distribution width [RDW] ≥ 15% (yes = 1, no = 0); best = 0, worst = 4) [5,6]. It was validated in the external cohort REALITY-AHF [7], which can reliably correlate time to treatment and clinical outcomes among the divergent group of HF patients admitted to the emergency department (ED) [8,9]. In the current study, we assessed the potential associations between the mode of death in HF and the LHFRS in the population of patients enrolled in the REALITY-AHF study.

## 2. Methods

### 2.1. Study Population

The REALITY-AHF (Registry Focused on Very Early Presentation and Treatment in Emergency Department of Acute Heart Failure) was a prospective, multicenter, observational cohort study that primarily aimed to assess the association between time to treatment and clinical outcomes in patients with acute HF (AHF) admitted through the emergency department (ED). Enrollment started in August 2014 and was completed in December 2015. Among the 20 participating hospitals, 9 were university hospitals and 11 were non-university teaching hospitals.

The study design and results have been reported elsewhere in detail [8,9,10]. In brief, patients were included if they were aged ≥20 years and diagnosed with AHF in the ED within 3 h of their first evaluation by caregivers. Only the first hospitalization during the study period was registered, and the AHF diagnosis was made based on the Framingham criteria. Exclusion criteria were as follows: (1) treatment with an intravenous (IV) drug before ED arrival; (2) previous heart transplantation; (3) chronic peritoneal dialysis or hemodialysis; (4) acute myocarditis; and (5) acute coronary syndrome requiring emergent or urgent revascularization. The study complied with the 1975 Declaration of Helsinki, and the Institutional Review Board (IRB) approval was obtained from each participating center.

In this study, we enrolled 1363 patients for whom LHFRS data were available.

### 2.2. Definitions

The red blood cell distribution width (RDW) was calculated as follows: (standard deviation of mean corpuscular volume divided by mean corpuscular volume) × 100. For an event causing death, the event and death were considered separate events only if the interval that separated the event and the death was 24 h or greater. In cases where the event and death were separated by less than 24 h, death was the only adjudicated event. All deaths were considered cardiovascular unless a non-cardiovascular cause of death was established. Cardiovascular deaths included death due to HF, myocardial infarction, SCD, other cardiovascular causes (e.g., stroke and cardiovascular intervention), and presumed cardiovascular causes [10]. Death due to HF is defined as death occurring in the context of clinically worsening symptoms and/or signs of HF without the evidence of another cause of death: (1) New or increasing symptoms and/or signs of HF requiring the initiation of, or an increase in, treatment directed at HF or occurring in a subject already receiving treatment; (2) HF symptoms or signs requiring continuous i.v. therapy or oxygen administration; (3) confinement to bed entirely due to HF symptoms; (4) pulmonary edema is sufficient to cause tachypnea and distress not occurring in the context of myocardial infarction or as a consequence of an arrhythmia occurring in the absence of worsening HF; (5) cardiogenic shock not occurring in the context of myocardial infarction or as a consequence of an arrhythmia occurring in the absence of worsening HF. In the current analysis, all-cause death was divided into cardiovascular and non-cardiovascular diseases. Cardiovascular death was divided into HF death, SCD, and other cardiovascular deaths.

### 2.3. Outcomes

The outcomes of interest were as follows: (a) SCD (primary endpoint); (b) death due to HF (secondary endpoint); (c) cardiovascular death (secondary endpoint); and (d) non-cardiovascular death (secondary endpoint), within 1-year after discharge.

### 2.4. Statistical Analysis

Categorical variables are shown as numbers and percentages and were compared using the Chi-squared test or Fisher’s exact test, as appropriate. Continuous variables were expressed as mean and standard deviation or median and interquartile range (IQR). Depending on their distribution (qualitatively judged via histogram and Q-Q plot), continuous variables were compared using Student’s *t*-test or Wilcoxon rank-sum test, as appropriate. Two-sided *p* values < 0.05 were considered statistically significant. Cumulative incidence curves were generated using a Fine–Gray competing risk regression, with deaths that were not due to the cause of death of interest as a competing risk. Likewise, the Fine–Gray competing risk regression analysis was used to evaluate the association between each variable and the incidence of each cause of death. Clinical follow-up data were obtained from medical records or directly from patients either in person or during telephone interviews. We used the AHEAD score for risk adjustment, which is a well-validated HF risk score ranging from 0 to 5 and includes the following variables: atrial fibrillation, anemia (haemoglobin <130 g/l for men and 120 g/l for women), age >70 years), renal dysfunction (creatinine >130 μmol/l), and diabetes mellitus [11,12]. Proportional hazard assumption violations were estimated using generalized linear regression of scaled Schoenfeld residuals over time. Statistical analyses were performed using the statistical software program R version 4.1.3 (R Foundation for Statistical Computing, Vienna, Austria).

## 3. Results

### 3.1. Baseline Characteristics

The baseline characteristics of the study population (1363 HF patients) stratified by the LHFRS score are presented in Table 1. Patients with higher LHFRS scores (i.e., 2–4) were younger and had lower admission systolic or diastolic blood pressure compared to patients with lower LHFRS (i.e., 0–1) scores. Additionally, they had a lesser history of hypertension and lower values of hemoglobin, white blood cells, glucose, and sodium. In contrast, patients with LHFRS 0 or 1 had a lesser history of coronary artery disease and lower values of RDW than those with LHFRS 2–4. Regarding medical therapy at admission, beta-blockers, mineralocorticoid receptor antagonists (MRAs), and loop diuretics were more frequently noted in the higher LHFRS categories, whereas angiotensin-receptor blockers (ARBs) were noted in the lower LHFRS categories.

### 3.2. Study Outcomes

**Figure 1:**
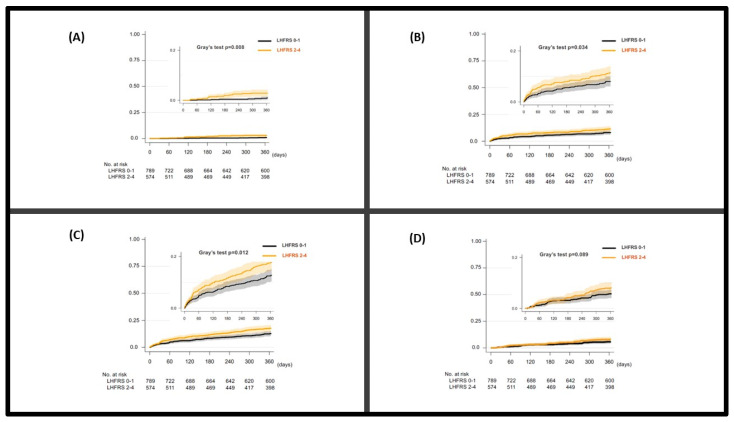
Cumulative incidence curves for (**A**) sudden cardiac death (SCD), (**B**) heart failure (HF) death, (**C**) cardiovascular (CV) death, and (**D**) non-cardiovascular (non-CV) death, based on the Larissa Heart Failure Risk Score (LHFRS).

During a median follow-up of 1 year, 284 deaths were observed: SCD (*n* = 23), heart failure deaths (*n* = 125), other cardiovascular deaths (*n* = 48), and non-cardiovascular deaths (*n* = 88). Patients with LHFRS 2–4 exhibited a significantly higher risk of SCD compared to those with LHFRS 0,1 (HR hazard ratio adjusted for AHEAD score 3.15, 95% confidence interval (CI) (1.30–7.65), *p* = 0.011) (Figure 1A, Table 2). The results were similar when medical treatment at discharge and estimated glomerular filtration rate (eGFR) were used also for risk adjustment (Please see Supplementary Table S1). Patients with LHFRS 2–4 demonstrated a significantly higher risk of HF death compared to those with LHFRS 0,1 (adjusted HR for AHEAD score 1.48, 95% CI (1.04–2.09), *p* = 0.03) (Figure 1B, Table 2).

Regarding cardiovascular death, patients with higher LHFRS had a significantly increased risk compared to those with lower LHFRS (HR 1.44 adjusted for AHEAD score, 95% CI (1.09–1.91), *p* = 0.01) (Figure 1C, Table 2). Lastly, patients with higher LHFRS exhibited a numerically higher risk of non-cardiovascular death compared to those with lower LHFRS, but it did not reach a statistically significant threshold (HR 1.44 adjusted for AHEAD score, 95% CI (0.95–2.19), *p* = 0.087) (Figure 1D, Table 2). LVEF did not modify the association between high/low LHFRS scores and the study outcomes (all *p* values for interaction were >0.05).

## 4. Discussion

In the REALITY-AHF trial, patients with AHF admitted to the ED were studied concerning the time of the first administration of IV diuretics and its clinical implication. A time-to-treatment benefit was observed, as patients with early diuretic administration (<60 min) demonstrated significantly lower in-hospital mortality [8,10]. The LHFRS was validated in the REALITY-AHF as an independent predictor of the primary and secondary outcomes of all-cause mortality and HF readmission [7]. In the present study, the cause of death was adjudicated according to the score’s three very specific variables: history of hypertension, history of coronary artery disease/myocardial infarction, and RDW value. A higher risk of SCD, HF death, and a significantly increased risk of cardiovascular death in the groups with higher LHFRS (2–4) highlighted those in greater hazard.

In the present analysis, we noticed that in the higher (2–4) group, a significant proportion of patients (27.6%) had preserved left ventricular ejection fraction (LVEF), whereas 32.3% of patients had LVEF <35%. This study population, which also encompasses patients with lower mean age, is a significant peril of SCD [13]. Since previous trials and risk scores using LVEF as a discriminator failed to demonstrate a significant benefit in patients with preserved LVEF, a notable proportion of HF patients forfeit treatment for SCD prevention, such as sacubitril-valsartan [14]. In the present work, we observed an independent association between the LHFRS and the mode of death, without considering LVEF, and provided a larger group of patients with prompt and appropriate medical care [15,16]. Since arrhythmic death is the cause in the majority of cases with SCD, patients in the aforementioned group would benefit from SCD reduction approaches, such as ventricular ectopy invigilation during their hospitalization and outpatients. A recent meta-analysis of seven randomized clinical trials, including patients with HF and reduced or preserved LVEF, revealed an association between the use of sodium-glucose transport protein 2 (SGLT2) inhibitors and reduced risk of SCD (risk ratios: 0.68; 95% [CI]: 0.48–0.95; *p* = 0.03; I^2^ = 0%) [17].

Another differentiating characteristic between LHFRS and the majority of the rest risk scores in HF is the ability of the first to reveal “high risk” AHF patients at the time of initial hospitalization, whereas most other models were applied to patients with chronic HF. The Seattle Heart Failure Model (SHFM) provides information about the likely mode of death among ambulatory HF patients, and the prognostication of the mode of death in PARAGON was evaluated in a group of patients with chronic HF [18,19]. Likewise, the evaluation of the mode of death in the PARADIGM-HF patients was achieved not on a single time point but integrated baseline characteristics as well as covariates that were collected from outpatient visits [20]. Similarly, CHARM, GISSI-HF, and MAGGIC prediction scores were performed in ambulatory HF patients [21]. The Metabolic Exercise test data combined with Cardiac and Kidney Indexes (MECKI) score is an established risk model in patients with systolic HF (i.e., LVEF < 40%) consisting of six variables: hemoglobin, serum sodium, kidney function by means of modification of diet in renal disease (MDRD), echocardiographic left ventricle ejection fraction, peak oxygen consumption (% predicted) and VE/VCO2 slope [22]. It was developed 10 years ago based on 2715 HF patients recruited and prospectively followed in 13 Italian HF centers and demonstrated excellent predictive value for the combined endpoint of death or heart transplantation with an area under the curve (AUC) ranging from 0.80 for events occurring within one year to 0.76 for events occurring within four years [23]. The MECKI score has been validated in different populations and has been proven to be a simple, practical tool for risk stratification in HF patients.

The contribution of risk stratification models in clinical practice is principal since they can change the trajectory of the disease by providing the option of timely therapy. However, their applicability in daily clinical practice has not been established, as most physicians find their use challenging [24,25]. The LHFRS is an easily obtainable risk stratification model since it consists of only three variables, which are typically obtained early at every admission in the ED. Other prognostic risk models have also been established in AHF, such as the Organized Program to Initiate Lifesaving Treatment in Hospitalized Patients with Heart Failure (OPTIMIZE-HF) scoring system [26], the Acute Decompensated Heart failure/N-Terminal proB-type Natriuretic Peptide (ADHF/NT-proBNP) risk score [27,28], the Acute Physiology and Chronic Health Evaluation-Heart Failure (APACHE-HF) scoring system [29] and the Evaluation Study of Congestive Heart Failure and Pulmonary Artery Catheterization Effectiveness (ESCAPE) discharge model [30]. The OPTIMIZE-HF scoring system is a useful bedside tool that includes the following eight variables: age, weight, systolic blood pressure, sodium, creatinine, history of liver disease, history of depression, and history of reactive airway disease. The scoring system has been utilized for the prediction of mortality risk (C-index of 0.72) in hospitalized HF patients within 60 days after their discharge [26]. The ADHF/NT-proBNP risk score contains a total of eight variables (chronic obstructive pulmonary disease, systolic blood pressure, eGFR, serum sodium, hemoglobin, NT-proBNP, LVEF, and tricuspid regurgitation moderate to severe), and the possible total score ranges from 0 to 22 [27]. The ADHF/NT-proBNP score exhibited an excellent discriminative ability for the endpoint of 1-year mortality (C index of 0.839) in a cohort of 453 ADHF patients (derivation cohort) and was successfully validated (C index of 0.768) in a cohort of 371 ADHF patients (validation cohort) [27]. The ADHF/NT-proBNP score has also been reported to predict 1-year mortality in 445 hospitalized advanced HF patients [28]. The APACHE- HF scoring system (mean arterial pressure, pulse, serum sodium, serum potassium, hematocrit, serum creatinine, age, and Glasgow Coma Scale) was found to be reliable in predicting adverse outcomes in 824 AHF patients and outperformed the more complex APACHE II (body temperature, mean blood pressure, pulse, respiratory rate, A-a DO2 (FiO2 ≥ 0.5), PaO2 (FiO2 < 0.5), arterial blood, serum sodium, serum potassium, hematocrit, creatinine, white blood cells, and Glasgow Coma Scale), as well as the modified APACHE II scoring system (age, mean blood pressure, pulse, serum sodium, serum potassium, serum creatinine, and Glasgow Coma Scale) [29]. The ESCAPE discharge risk model (age, blood urine nitrogen, 6 min walking test, sodium, cardiopulmonary resuscitation/mechanical ventilation, diuretic dose, no beta-blocker at discharge, discharge brain natriuretic peptide (BNP)) has been shown to predict the risk of death at 6 months (C-index of 0.739) in a cohort of 423 patients with advanced decompensated systolic HF [30]. However, the majority of these risk models are either complicated, using many variables, or limited to systolic HF groups.

Among the comparison of major HF risk models, including CHARM, MAGGIC, GISSI-HF, and SHFM, MAGGIC showed the best overall accuracy in predicting one-year mortality, using 11 variables, whereas SHFM, being the most sophisticated, using 24 variables, demonstrated a lower overestimation of mortality [18,21,31]. On the other hand, the use of oversimplified risk scores may be of doubtful clinical value. Thus, the use of bilirubin level as a discriminator in PRAISE (The Prospective Randomized Amlodipine Evaluation Study) cohort patients signified an increased risk of pump failure death but failed to detect those in danger of SCD [32].

## 5. Strengths and Limitations

The LHFRS was validated in the REALITY-HF patient population and applied to hospitalized patients only. Certainly, in the trajectory of HF, emerging biomarkers, such as electrolyte disturbances, hypoalbuminemia, and hyperuricemia, can significantly influence the disease outcome and should be monitored in an extended timeline. However, previous analyses have been mainly limited to chronic HF patients. Another substantial limitation is that, due to the disunity of HF pathophysiology, at present LHFRS cannot be used to guide treatment in AHF. Lastly, patients participating in the REALITY-AHF were not on the more recently approved life-prolonging HF drugs (endorsed by international guidelines), such as angiotensin receptor neprilysin inhibitor (ARNI) or SGLT2 inhibitors [33,34], since enrollment of these patients took place earlier (2014–2015); therefore, these drugs were not available. Despite these limitations, the present work demonstrates, for the first time, the independent association of simple LHFRS with SCD and death from heart (pump) failure in a “real world” cohort of hospitalized patients with AHF. In this regard, a high LHFRS score may identify patients at a greater risk of SCD, HF, or cardiovascular death and orientate them to close monitoring in established HF centers.

## 6. Conclusions

Increased LHFRS was independently associated with SCD and HF death in a prospective cohort of AHF patients.

## Figures and Tables

**Table 1:** Baseline characteristics of the study population.

Variables	LHFRS = 0, 1 *n* = 789	LHFRS = 2–4 *n* = 574	*p* Value
Age (mean (SD))	78.47 (12.10)	75.56 (12.90)	<0.001
Males, *n* (%)	430 (54.5)	339 (59.1)	0.105
Systolic Blood Pressure (mean (SD))	154.90 (35.67)	135.47 (33.20)	<0.001
Diastolic Blood Pressure (mean (SD))	85.74 (25.67)	78.86 (22.63)	<0.001
Heart Rate (mean (SD))	96.98 (27.80)	96.56 (28.48)	0.784
Ambulance, *n* (%)	452 (57.3)	306 (53.3)	0.16
De novo HF, *n* (%)	400 (50.7)	247 (43.0)	0.006
Symptom onset (%)			0.013
6 h	188 (23.8)	108 (18.8)	
≤2 days	190 (24.1)	122 (21.3)	
>2 days	411 (52.1)	344 (59.9)	
ECG rhythm (%)			0.001
Sinus rhythm	446 (56.5)	268 (46.7)	
Atrial fibrillation	262 (33.2)	239 (41.6)	
Other	81 (10.3)	67 (11.7)	
Echo visual estimation of LVEF (%)			<0.001
<35	243 (32.3)	256 (46.7)	
35–50	228 (30.3)	141 (25.7)	
>50	281 (37.4)	151 (27.6)	
Heart Failure Symptoms/Signs			
Jugular Venous Distension, *n* (%)	428 (54.7)	319 (56.2)	0.623
Orthopnea, *n* (%)	471 (59.8)	290 (50.5)	0.001
Rales, *n* (%)	512 (64.9)	355 (62.0)	0.291
Peripheral edema, *n* (%)	531 (67.3)	393 (68.6)	0.658
Pulmonary edema, *n* (%)	594 (75.3)	378 (65.9)	<0.001
Comorbidities/Risk factors			
Hypertension, *n* (%)	789 (100.0)	131 (22.8)	<0.001
Diabetes Mellitus, *n* (%)	305 (38.7)	193 (33.6)	0.065
Coronary Artery Disease, *n* (%)	213 (27.0)	224 (39.0)	<0.001
Peripheral Arterial Disease, *n* (%)	64 (8.1)	39 (6.8)	0.421
Chronic Obstructive Pulmonary Disease, *n* (%)	78 (9.9)	53 (9.2)	0.756
Smoker, *n* (%)	287 (36.4)	212 (37.0)	0.858
Laboratory Variables			
Hemoglobin (mean (SD))	11.83 (2.23)	11.56 (2.36)	0.033
RDW-CV (mean (SD))	14.56 (1.64)	15.56 (2.30)	<0.001
White Blood Cells (median [IQR])	7800 [5900, 10,400]	7000 [5500, 9300]	<0.001
Glucose (mean (SD))	166.65 (75.96)	157.20 (76.26)	0.026
Blood Urine Nitrogen (median [IQR])	24.50 [17.80, 34.60]	25 [18.42, 36]	0.288
Creatinine (median [IQR])	1.13 [0.86, 1.64]	1.12 [0.85, 1.58]	0.585
Estimated Glomerular Filtration Rate (mean (SD))	55.17 (25.14)	58.00 (26.22)	0.044
Aspartate Aminotransferase (median [IQR])	30 [23, 44]	33 [23, 49]	0.068
Alanine Aminotransferase (median [IQR])	21 [14, 34]	22 [14, 37]	0.286
Na^+^ (mean (SD))	139.20 (4.61)	138.28 (4.44)	<0.001
CRP (median [IQR])	0.58 [0.19, 2.26]	0.75 [0.22, 2.04]	0.175
Medications at admission			
ACE-inhibitors, *n* (%)	135 (17.1)	99 (17.2)	1
Angiotensin Receptor Blockers, *n* (%)	296 (37.5)	121 (21.1)	<0.001
Beta Blockers, *n* (%)	330 (42.0)	280 (49.0)	0.013
Mineralocorticoid Antagonists, *n* (%)	131 (16.6)	171 (29.8)	<0.001
Loop diuretics, *n* (%)	376 (48.1)	332 (57.9)	<0.001
Medications at discharge			
ACE-inhibitors, *n* (%)	246 (32.8)	193 (36.2)	0.220
Angiotensin Receptor Blockers, *n* (%)	284 (37.8)	119 (22.3)	<0.001
Beta Blockers, *n* (%)	546 (72.4)	395 (73.8)	0.616
Mineralocorticoid Antagonists, *n* (%)	318 (42.1)	264 (49.3)	0.012
Loop diuretics, *n* (%)	640 (84.7)	460 (85.7)	0.674

LVEF, left ventricular ejection fraction; RDW, red blood cell distribution width; CRP, C-reactive protein. ECG: electrocardiogram, LVEF: left ventricular ejection fraction, RDW: red blood cell distribution width, Na^+^: sodium, CRP: C-reactive protein, ACE-inhibitors: angiotensin converting enzyme inhibitors

**Table 2:** Comparison of risk (unadjusted and adjusted for the AHEAD score) of sudden cardiac death (SCD), heart failure (HF) death, cardiovascular (CV) death, and non-cardiovascular (non-CV) death, in patients with LHFRS 0, 1 vs. LHFRS 2–4.

Sudden Cardiac Death						
Groups	Unadjusted			Adjusted for AHEAD Score		
	HR	95% CI	*p* Value	HR	95% CI	*p* Value
AHEAD score	1.33	1.02–1.72	0.033	1.23	0.93–1.63	0.15
LARISSA Score 0,1		1 (Reference)			1 (Reference)	
LARISSA Score 2–4	3.14	1.29–7.61	0.008	3.15	1.30–7.65	0.011
Heart Failure Death						
Groups	Unadjusted			Adjusted for AHEAD score		
	**HR**	95% CI	*p* value	HR	95% CI	*p* value
AHEAD score	1.43	1.24–1.64	<0.001	1.38	1.18–1.60	<0.001
LARISSA Score 0,1		1 (Reference)			1 (Reference)	
LARISSA Score 2–4	1.46	1.03–2.07	0.034	1.48	1.04–2.09	0.03
Cardiovascular Death						
Groups	Unadjusted			Adjusted for AHEAD score		
	**HR**	95% CI	*p* value	HR	95% CI	*p* value
AHEAD score	1.38	1.23–1.54	<0.001	1.32	1.17–1.49	<0.001
LARISSA Score 0,1		1 (Reference)			1 (Reference)	
LARISSA Score 2–4	1.43	1.08–1.89	0.012	1.44	1.09–1.91	0.01
Non-cardiovascular Death						
Groups	Unadjusted			Adjusted for AHEAD score		
	**HR**	95% CI	*p* value	HR	95% CI	*p* value
AHEAD score	1.26	1.09–1.47	0.002	1.28	1.13–1.42	0.003
LARISSA Score 0,1		1 (Reference)			1 (Reference)	
LARISSA Score 2–4	1.44	0.95–2.18	0.089	1.44	0.95–2.19	0.087

## Data Availability

The registry focused on very early presentation and treatment in the emergency department of acute heart failure syndrome (REALITY-AHF) (UMIN000014105).

## Supplementary Materials

The following supporting information can be downloaded at: https://www.mdpi.com/article/10.3390/jcm12113722/s1, Table S1. Comparison of risk of sudden cardiac death (SCD) in patients with LHFRS 0,1 vs. LHFRS 2–4.
