# Supplementary material for: Larissa Heart Failure Risk Score and Mode of Death in Acute Heart Failure: Insights from REALITY-AHF
Source: J Clin Med. 2023 May 28;12(11):3722. doi: 10.3390/jcm12113722 (PMC10253707; doi:10.3390/jcm12113722)
Supplement: Supplementary file 1 [file jcm-12-03722-s001.zip › jcm-2269750-supplementary.pdf]

Suppl. Material

Larissa Heart Failure Risk Score and Mode of Death in Acute Heart Failure: Insights From REALITY-AHF

Suppl. Table S1. Comparison of risk of sudden cardiac death (SCD) in patients with LHFRS 0,1 vs. LHFRS 2-4.

**Sudden Cardiac Death**			
Groups	Adjusted for AHEAD and Angiotensin Receptor Blockers<br><br>HR	95% CI	P value
**LARISSA Score 0,1**		1 (Reference)	
**LARISSA Score 2-4**	3.13	1.21-8.13	**0.019**
	Adjusted for AHEAD and Mineralocorticoid Antagonists<br><br>HR	95% CI	P value
**LARISSA Score 0,1**		1 (Reference)	
**LARISSA Score 2-4**	3.74	1.47-9.50	**0.006**
	Adjusted for AHEAD and eGFR		
**LARISSA Score 0,1**		1 (Reference)	
**LARISSA Score 2-4**	3.19	1.32-7.71	**0.01**

Patients with LHFRS 2-4 exhibited significantly higher risk for sudden cardiac death (primary endpoint) compared to those with LHFRS 0,1 after adjustment for AHEAD and Angiotensin Receptor Blockers, AHEAD and Mineralocorticoid Antagonists, AHEAD and eGFR. Parameters were carefully selected to avoid overfitting.
